# Draft Genome Sequence of Clostridium tagluense Strain A121^T^, Isolated from a Permafrost Core in the Canadian High Arctic

**DOI:** 10.1128/MRA.01630-18

**Published:** 2019-01-31

**Authors:** Takumi Murakami, Hiroshi Mori, Viktoria A. Shcherbakova, Yoshitaka Yoshimura, Takahiro Segawa

**Affiliations:** a Center for Information Biology, National Institute of Genetics, Mishima, Shizuoka, Japan; b Skryabin Institute of Biochemistry and Physiology of Microorganisms, Russian Academy of Sciences, Pushchino, Moscow Region, Russia; c College of Agriculture, Tamagawa University, Machida, Tokyo, Japan; d Center for Life Science Research, University of Yamanashi, Chuo, Yamanashi, Japan; e National Institute of Polar Research, Tachikawa, Tokyo, Japan; University of Arizona

## Abstract

Clostridium tagluense strain A121^T^ was isolated from a permafrost core in the Canadian High Arctic. This endospore-forming strain is a strictly anaerobic, Gram-positive, and psychrotolerant bacterium. This article describes the draft genome sequence of C. tagluense strain A121^T^.

## Abstract (precis)

Clostridium tagluense strain A121^T^ was isolated from a permafrost core in the Canadian High Arctic. This endospore-forming strain is a strictly anaerobic, Gram-positive, and psychrotolerant bacterium.

## ANNOUNCEMENT

Clostridium tagluense strain A121^T^ (VKM B-2369^T^ = DSM 17763^T^) is a strictly anaerobic, Gram-positive, motile, and endospore-forming bacterium isolated from a Quaternary-aged permafrost sample with a temperature of −6°C that was collected from the upper horizon (18 m) of the core of the deep (451 m) borehole 92GSCTaglu in a gas-hydrate field in the Mackenzie Delta of the Northwest Territories in Canada (1). The previous phylogenetic analysis based on 16S rRNA gene sequences indicated that strain A121^T^ is affiliated with a clade of psychrophilic and psychrotolerant *Clostridium* species (1). In fact, strain A121^T^ is a psychrotolerant bacterium, which can grow at temperatures as low as 4°C with an optimum temperature of 15–20°C (1).

Strain A121^T^ was cultivated as described previously (1). Briefly, bacterial cells were enriched under anaerobic conditions at 6°C, and then aliquots of the enrichment culture were plated on agar plates to isolate single colonies. The pure culture of strain A121^T^ was maintained in peptone yeast (PY) broth at 15°C. Genomic DNA was extracted with a DNeasy UltraClean microbial kit (Qiagen) and sheared to approximately 450 bp with an M220 instrument (Covaris). A DNA library was constructed with a KAPA HyperPrep kit PCR-free (Roche) and sequenced on a MiSeq platform (Illumina) with its reagent kit v2 (2 × 250 bp paired-end protocol). The obtained reads were assembled with SPAdes v3.10.1 (2) with the careful mode enabled.

A total of 5,457,647 paired-end reads were assembled into 168 scaffolds with 350× coverage, the largest length being 331,140 bp, and an *N*_50_ value of 132,742 bp. The draft genome sequence is composed of 5,195,089 bp, with a GC content of 30.9%. A total of 4,935 protein-coding sequences and 17 rRNA genes were predicted using the DFAST server (3). The genome of strain A121^T^ encodes four genes for cold shock proteins, which would act as chaperones for mRNA during cold shock (4). It also encodes a set of genes for an osmoprotectant ABC transporter (OpuBA, OpuBB, and OpuBC), which may contribute to resistance against osmotic stress in cold environments (5).

The genome sequence reported here will promote further studies of the physiology and genomics of C. tagluense strain A121^T^ and the related psychrophilic and psychrotolerant *Clostridium* species. Analysis of the genomic data will also allow us to understand the molecular mechanisms of the bacterial adaptation to low and subzero temperatures.

### Data availability.

The genome sequence of Clostridium tagluense strain A121^T^ and corresponding raw sequence data have been deposited at DDBJ/ENA/GenBank under the accession no. BHYK01000001 to BHYK01000168 and in the Sequence Read Archive under accession no. DRA007509.
